# Supplementary material for: Deep terrestrial indigenous microbial community dominated by Candidatus Frackibacter
Source: Commun Earth Environ. 2024 Dec 29;5(1):795. doi: 10.1038/s43247-024-01966-8 (PMC11683007; doi:10.1038/s43247-024-01966-8)
Supplement: Supplementary file 2 — Supplemental Material [file 43247_2024_1966_MOESM2_ESM.pdf]

Supplemental Table S1. Geochemistry of Kidd Creek Borehole Fluids and Service Water.

Date	January 24-25 2017			June 06-07 2017			January 28-29 2018		
Sample	Service Water	FW12261	FW12299	Service Water	FW12261	FW12299	Service Water	FW12261	FW12299
$\delta^{13}C_{CH4}$ (‰)	NR	-39.6	-38.5	NR	-38.5	-39.1	NR	-38.5	-39.0
$\delta^{18}O_{H2O}$(‰)	-10.6	-12.4	-12.4	-12.4	-11.6	-11.1	-10.7	NM	-12.4
$\delta^{2}H_{H2O}$(‰)	-80.8	-33.6	-34.2	-89.8	-33.9	-34.1	-83.1	NM	-35.5
Li^{+} (μg/L)	<500	<500	616	<500	<500	636	<500	NM	<5000
Na^{+} (mg/L)	377	17300	15300	194	17900	17300	261	NM	14900
K^{+} (mg/L)	53	108	100	25	118	123	54	NM	195
Mg^{2+} (mg/L)	106	2020	2220	76	2120	2460	52	NM	2110
Ca^{2+} (mg/L)	422	65500	49700	350	68300	58200	322	NM	50800
Cl^{-} (mg/L)	252	136000	115000	201	120000	110000	232	NM	125000
Br^{-} (mg/L)	3	2000	1730	2	1910	1760	3	NM	1890
NO^{3-} (mg/L)	18	<2	<2	15	<5	<5	6	NM	<20
NO^{2-} (mg/L)	2	<2	<2	<0.1	<5	<5	<0.2	NM	<20
HPO_4^{2-} (mg/L)	<0.2	<4	<4	<0.2	<10	<10	<0.4	NM	<30
SO_4^{2-} (mg/L)	1930	20	25	1300	10	10	1300	NM	<40
Mn (μg/L)	3370	33400	24900	2500	33900	28700	1100	NM	22100
Fe (mg/L)	<5	26	35	<5	17	16	1	NM	10
Zn (μg/L)	36900	<250	<250	29500	<250	<250	7400	NM	<500
Ba (μg/L)	<50	43800	41700	<50	43300	42900	<200	NM	32700
B (μg/L)	<1500	3140	2880	<1500	3450	3540	NM	NM	NM
Sr (mg/L)	4	1200	NM	3	1100	997	3	NM	971
Total Dissolved Solids (mg/L)	5125	225235	185062	4042	212552	191887	2520	NM	197274

NM - not measured NR - not relevant

Supplemental Table S2. Borehole and service water conditions.

Sample	FW12261 2017A	FW12261 2017B	FW12261 2018	FW12299 2017A	FW12299 2017B	FW12299 2018	Biofilm A	Biofilm B	Service Water 2018
Borehole	12261	12261	12261	12299	12299	12299	12261	12261	NR
Date Installed	Jul 12 2016	Jul 12 2016	Jun 06 2017	Jan 24 2017	Jan 24 2017	Jun 06 2017	NR	NR	NR
Date Collected	Jan 24 2017	Jan 24 2017	Jan 28 2018	Jun 06 2017	Jun 06 2017	Jan 28 2018	July 12 2016	Jun 06 2017	Jan 28 2018
Deployment Duration (days)	196	196	236	133	133	236	NR	NR	NR
Flow rate at installation (mL/min)	NM	NM	Gas: 2729 Water: 134	Gas: 3082 Water: 280	Gas: 3082 Water: 280	Gas: 2729 Water: 134	NR	NR	NR
Flow rate at removal (mL/min)	Gas: 3082 Water: 280	Gas: 3082 Water: 280	Gas: 5938 Water: 127	Gas: 2729 Water: 134	Gas: 2729 Water: 134	Gas: 5938 Water: 127	NR	NR	NR
Average Temperature (°C)	25	25	25	24.5	24.5	24.5	25	25	23.6
Average pH	6.3	6.3	6.3	6.7	6.7	6.7	6.3	6.3	7.2

NM - not measured NR - not relevant

Supplemental Table S3. Relative abundance (mole %) of each PLFA detected in all biosamplers, biofilms and service water samples. Where values are absent, PLFA was below the limit of quantification (LoQ < 0.50 µg/mL).

PLFA	FW12261 2017A	FW12261 2017B	FW12261 2018	FW12288 2017A	FW12288 2017B	FW12288 2018	Biofilm 2016	Biofilm 2017	Service Water 2018
12:0	0.4		3	11	26	6			
13:0				1	0.3				
14:0	2	1	3	5	7	3	1	1	1
15:0	0.3	0.2	1	0.2	0.1	0.3	2	3	1
16:0	26	12	26	24	29	53	41	50	22
17:0	0.4	0.2	1	0.2	0.3	1	2	2	0.5
18:0	8	13	11	30	23	20	5	5	1
19:0								0.2	
20:0			0.5	0.01	0.3	1		0.1	
22:0			0.4						
23:0						1			
24:0						1			
$14:1^{x}$	0.1								
$15:1^{x}$	0.1	0.1		3	1				1
$16:1^{x}$	5	8	1	0.3		0.2	0.4	1	49
$16:1^{9}$			4	1	1	1	5	5	
$17:1^{x}$	0.1	0.2	0.4			0.3			0.3
$17:1^{10}$			1				2	1	
$18:1^{9}c$	8	13	0	4	4	9	33	16	2
$18:1^{9}t$	8	13	34	1	1	2	2	4	17
$18:1^{x}$	0.3	0.1	3						
$19:1^{x}$							0.1		1
$20:1^{x}$	0.004	0.1						0.1	
$18:3^{x,y,z}$									0.3
$18:2c^{9,12}$	1	1		0.2	0.3	1	3	2	0.4
$18:2^{x,y}$			8						
br14:0								0.1	
br15:0				6					

i15:0	0.5	0.3	2	7	3	0.3	1	4	1
a15:0			1			0.2	0.2	1	1
br16:0	0.3			1	1				
i16:0	0.05	0.2		1				0.2	0.3
a16:0				0.1	0.2				0.4
br17:0				3	1		0.4	1	
i17:0	0.1	0.1		0.2	0.2		0.1	0.3	
a17:0				0.2	0.2				
br18:0	0.1								
br19:0	0.02								
br20:0	0.2	0.04	0.5						
cy17:0Δ^9c	14	14	1						3
cy17:0Δ^x	0.6	0.3							
cy19:0Δ^x		2							
cy19:0Δ^9c	24	21	1				3	2	
dibromo-18:0								0.2	
