# Supplementary material for: Deep terrestrial indigenous microbial community dominated by Candidatus Frackibacter
Source: Commun Earth Environ. 2024 Dec 29;5(1):795. doi: 10.1038/s43247-024-01966-8 (PMC11683007; doi:10.1038/s43247-024-01966-8)
Supplement: Supplementary file 3 — Reporting Summary [file 43247_2024_1966_MOESM3_ESM.pdf]

nature portfolio

Corresponding author(s): Greg Slater : gslater@mcmaster.ca

Last updated by author(s): Dec 12, 2024

Reporting Summary

Nature Portfolio wishes to improve the reproducibility of the work that we publish. This form provides structure for consistency and transparency in reporting. For further information on Nature Portfolio policies, see our Editorial Policies and the Editorial Policy Checklist.

Statistics

For all statistical analyses, confirm that the following items are present in the figure legend, table legend, main text, or Methods section.

n/a	Confirmed	
☐	☒	The exact sample size (*n*) for each experimental group/condition, given as a discrete number and unit of measurement
☐	☒	A statement on whether measurements were taken from distinct samples or whether the same sample was measured repeatedly
☐	☒	The statistical test(s) used AND whether they are one- or two-sided<br>*Only common tests should be described solely by name; describe more complex techniques in the Methods section.*
☒	☐	A description of all covariates tested
☐	☒	A description of any assumptions or corrections, such as tests of normality and adjustment for multiple comparisons
☐	☒	A full description of the statistical parameters including central tendency (e.g. means) or other basic estimates (e.g. regression coefficient) AND variation (e.g. standard deviation) or associated estimates of uncertainty (e.g. confidence intervals)
☒	☐	For null hypothesis testing, the test statistic (e.g. *F*, *t*, *r*) with confidence intervals, effect sizes, degrees of freedom and *P* value noted<br>*Give P values as exact values whenever suitable.*
☒	☐	For Bayesian analysis, information on the choice of priors and Markov chain Monte Carlo settings
☐	☒	For hierarchical and complex designs, identification of the appropriate level for tests and full reporting of outcomes
☐	☒	Estimates of effect sizes (e.g. Cohen's *d*, Pearson's *r*), indicating how they were calculated

Our web collection on statistics for biologists contains articles on many of the points above.

Software and code

Policy information about availability of computer code

Data collection	Gas chromatography and mass spectrometry data was collected using ChemStation
Data analysis	Data analysis was performed using Microsoft Excel and MetaboAnalyst Open Access analysis software. Genetic data was processed using QIIME2 (https://qiime2.org/) for the data analysis and Decontam (https://github.com/benjjneb/DecontamManuscript). Faprotax (https://pages.uoregon.edu/slouca/LoucaLab/archive/FAPROTAX/lib/php/index.php) was used for metabolic inferences as described in the text.

For manuscripts utilizing custom algorithms or software that are central to the research but not yet described in published literature, software must be made available to editors and reviewers. We strongly encourage code deposition in a community repository (e.g. GitHub). See the Nature Portfolio guidelines for submitting code & software for further information.

Data

Policy information about availability of data

All manuscripts must include a data availability statement. This statement should provide the following information, where applicable:

- Accession codes, unique identifiers, or web links for publicly available datasets
- A description of any restrictions on data availability
- For clinical datasets or third party data, please ensure that the statement adheres to our policy

The data for this project has been made available in the following repositories. Phospholipid fatty acid, stable carbon isotope, and geochemical data has been made

available in the EarthChem Library (https://doi.org/10.60520/IEDA/113522). 16S rRNA sequence data is available through the European Nucleotide Archive under accession numbers PRJEB49237.

Human research participants

Policy information about studies involving human research participants and Sex and Gender in Research.

Reporting on sex and gender	*Use the terms sex (biological attribute) and gender (shaped by social and cultural circumstances) carefully in order to avoid confusing both terms. Indicate if findings apply to only one sex or gender; describe whether sex and gender were considered in study design whether sex and/or gender was determined based on self-reporting or assigned and methods used. Provide in the source data disaggregated sex and gender data where this information has been collected, and consent has been obtained for sharing of individual-level data; provide overall numbers in this Reporting Summary. Please state if this information has not been collected. Report sex- and gender-based analyses where performed, justify reasons for lack of sex- and gender-based analysis.*
Population characteristics	*Describe the covariate-relevant population characteristics of the human research participants (e.g. age, genotypic information, past and current diagnosis and treatment categories). If you filled out the behavioural & social sciences study design questions and have nothing to add here, write "See above."*
Recruitment	*Describe how participants were recruited. Outline any potential self-selection bias or other biases that may be present and how these are likely to impact results.*
Ethics oversight	*Identify the organization(s) that approved the study protocol.*

Note that full information on the approval of the study protocol must also be provided in the manuscript.

Field-specific reporting

Please select the one below that is the best fit for your research. If you are not sure, read the appropriate sections before making your selection.

☐ Life sciences ☐ Behavioural & social sciences ☒ Ecological, evolutionary & environmental sciences

For a reference copy of the document with all sections, see nature.com/documents/nr-reporting-summary-flat.pdf

Ecological, evolutionary & environmental sciences study design

All studies must disclose on these points even when the disclosure is negative.

Study description	This study was an investigation of the microbial life living associated with ancient fracture waters within the deep continental subsurface. Phospholipid fatty acids were extracted and purified from biosampler material, microbial biofilms and service water and identified to create a unique profile for each sample. This data was analyzed using Spearman's rank correlation to reveal correlations between sample profiles. Samples were also extracted for 16S rRNA to identify the organisms present as well as their relative abundance. FAPROTAX was performed to help identify potential metabolic capabilities along with stable carbon analysis of the phospholipid fatty acids and potential carbon pools including dissolved inorganic carbon, acetate, formate and methane.
Research sample	Biosampler material, microbial biofilm, and service waters from the Kidd Creek Mine, taken in 2016, 2017, and 2018.
Sampling strategy	A series of biosamplers were used to passively collect microbial biomass from hyper-saline fracture waters flowing from two boreholes. Biofilms growing adjacent to one of the boreholes was also collected.
Data collection	Broadly, the data was collected by all co-authors. The main data sets included here were the phospholipid fatty acid and associated stable carbon data, which was collected by Sian E. Ford, and the 16S rRNA data which was collected by Katja Engels and Sian E. Ford.
Timing and spatial scale	Field work was performed on July 12 in 2016, January 24 and June 06 in 2017, and January 29 and July 20 in 2018. Samples were processed and analyzed between 2016 and 2019, following relevant field trips.
Data exclusions	No data were excluded from the analyses.
Reproducibility	Reproducibility of lab work was ensured by rigorous internal testing of wet lab SOPs. Reproducibility of data acquisition was ensured by running sufficient method controls, standards and standard mixtures during sample analysis.
Randomization	Randomization was not necessary as samples were all encompassing for the time point at which they were taken.
Blinding	Samples underwent analysis in a random order along with relevant controls, blanks and standards.

Did the study involve field work? ☒ Yes ☐ No

Field work, collection and transport

Field conditions	Field site was located 7850ft below surface level in an active mine environment. Ambient air temperature was approximately 25 degrees Celsius.
Location	Kidd Creek Observatory, Kidd Creek Mine, Timmins, Ontario, Canada. Latitude: 48.68. Longitude: -81.37
Access & import/export	The mine was accessed via permission of the land owners who gave permission for sample collection.
Disturbance	No disturbance was caused by the study.

Reporting for specific materials, systems and methods

We require information from authors about some types of materials, experimental systems and methods used in many studies. Here, indicate whether each material, system or method listed is relevant to your study. If you are not sure if a list item applies to your research, read the appropriate section before selecting a response.

Materials & experimental systems

n/a	Involved in the study
☒	☐ Antibodies
☒	☐ Eukaryotic cell lines
☒	☐ Palaeontology and archaeology
☒	☐ Animals and other organisms
☒	☐ Clinical data
☒	☐ Dual use research of concern

Methods

n/a	Involved in the study
☒	☐ ChIP-seq
☒	☐ Flow cytometry
☒	☐ MRI-based neuroimaging
